# 3-Cyclo­hexyl­sulfonyl-2,5-dimethyl-1-benzofuran

**DOI:** 10.1107/S160053681101498X

**Published:** 2011-04-29

**Authors:** Pil Ja Seo, Hong Dae Choi, Uk Lee

**Affiliations:** a Department of Chemistry, Dongeui University, San 24 Kaya-dong Busanjin-gu, Busan 614-714, Republic of Korea; b Department of Chemistry, Pukyong National University, 599-1 Daeyeon 3-dong, Nam-gu, Busan 608-737, Republic of Korea

## Abstract

In the title compound, C_16_H_20_O_3_S, the cyclo­hexyl ring adopts a chair conformation and the aryl­sulfonyl unit is in the equatorial position. In the crystal, mol­ecules are linked through weak inter­molecular C—H⋯O hydrogen bonds and C—H⋯π inter­actions.

## Related literature

For the pharmacological activity of benzofuran compounds, see: Aslam *et al.* (2009 ▶); Galal *et al.* (2009 ▶); Khan *et al.* (2005 ▶). For natural products with benzofuran rings, see: Akgul & Anil (2003 ▶); Soekamto *et al.* (2003 ▶). For structural studies of related 3-cyclo­hexyl­sulfonyl-2-methyl-1-benzofuran derivatives, see: Choi *et al.* (2011**a* ▶,b* ▶).
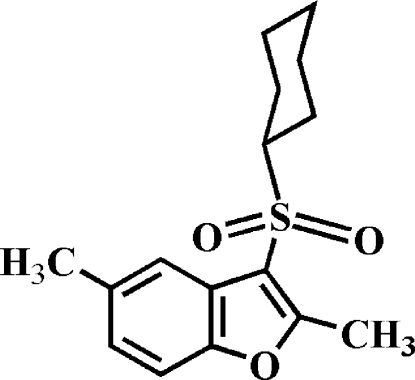


## Experimental

### 

#### Crystal data

C_16_H_20_O_3_S
*M* *_r_* = 292.38
Monoclinic, 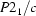

*a* = 5.6854 (3) Å
*b* = 21.2391 (13) Å
*c* = 12.3944 (7) Å
β = 99.295 (3)°
*V* = 1477.01 (15) Å^3^
*Z* = 4
Mo *K*α radiation
μ = 0.22 mm^−1^
*T* = 173 K
0.30 × 0.22 × 0.20 mm

#### Data collection

Bruker SMART APEXII CCD diffractometer
Absorption correction: multi-scan (*SADABS*; Bruker, 2009 ▶) *T*_min_ = 0.643, *T*_max_ = 0.746
13128 measured reflections
3228 independent reflections
2477 reflections with *I* > 2σ(*I*)
*R*_int_ = 0.035

#### Refinement

*R*[*F* ^2^ > 2σ(*F* ^2^)] = 0.040
*wR*(*F* ^2^) = 0.106
*S* = 1.05
3228 reflections
183 parameters
H-atom parameters constrained
Δρ_max_ = 0.32 e Å^−3^
Δρ_min_ = −0.34 e Å^−3^

### 

Data collection: *APEX2* (Bruker, 2009 ▶); cell refinement: *SAINT* (Bruker, 2009 ▶); data reduction: *SAINT* ; program(s) used to solve structure: *SHELXS97* (Sheldrick, 2008 ▶); program(s) used to refine structure: *SHELXL97* (Sheldrick, 2008 ▶); molecular graphics: *ORTEP-3* (Farrugia, 1997 ▶) and *DIAMOND* (Brandenburg, 1998 ▶); software used to prepare material for publication: *SHELXL97*.

## Supplementary Material

Crystal structure: contains datablocks global, I. DOI: 10.1107/S160053681101498X/gk2370sup1.cif

Structure factors: contains datablocks I. DOI: 10.1107/S160053681101498X/gk2370Isup2.hkl

Supplementary material file. DOI: 10.1107/S160053681101498X/gk2370Isup3.cml

Additional supplementary materials:  crystallographic information; 3D view; checkCIF report

## Figures and Tables

**Table 1:** Hydrogen-bond geometry (Å, °) *Cg* is the centroid of the C2–C7 benzene ring.

*D*—H⋯*A*	*D*—H	H⋯*A*	*D*⋯*A*	*D*—H⋯*A*
C11—H11⋯O2^i^	1.00	2.31	3.273 (2)	161
C12—H12*B*⋯O3^ii^	0.99	2.57	3.443 (2)	146
C10—H10*C*⋯*Cg*^iii^	0.99	2.75	3.556 (2)	140

Symmetry codes: (i) 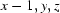; (ii) 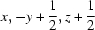; (iii) 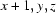.

**Fig. 1.**
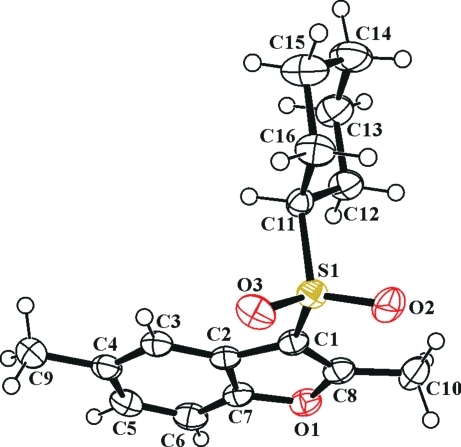
The molecular structure of the title compound. Displacement ellipsoids are drawn at the 50% probability level. H atoms are presented as small spheres of arbitrary radius.

**Fig. 2.**
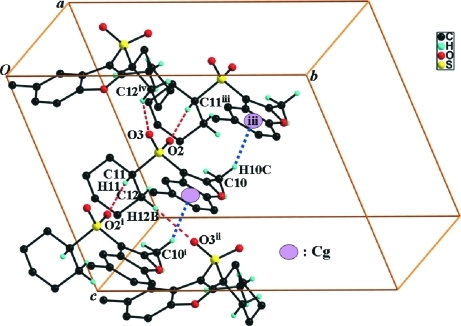
A view of the C–H···O and C–H···π interactions (dotted lines) in the crystal structure of the title compound. [Symmetry codes: (i) x - 1, y, z; (ii) x, - y + 1/2, z + 1/2; (iii) x + 1, y, z; (iv) x, - y + 1/2, z - 1/2.]

## supplementary crystallographic information

### Comment

Many compounds containing a benzofuran ring have drawn much attention owing to their diverse pharmacological properties such as antibacterial and antifungal, antitumor and antiviral, and antimicrobial activities (Aslam *et al.*, 2009, Galal *et al.*, 2009, Khan *et al.*, 2005). These compounds occur in a wide range of natural products (Akgul & Anil, 2003; Soekamto *et al.*, 2003). As a part of our ongoing study of the substituent effect on the solid state structures of 3-cyclohexylsulfonyl-2-methyl-1-benzofuran analogues (Choi *et al.*, 2011**a*,b*), we report herein the crystal structure of the title compound.

In the title molecule (Fig. 1), the benzofuran unit is essentially planar, with a mean deviation of 0.006 (1) Å from the least-squares plane defined by the nine constituent atoms. The cyclohexyl ring is in the chair form. The molecular packing (Fig. 2) is stabilized by weak intermolecular C–H···O hydrogen bonds; the first one between a cyclohexyl H atom and the O atom of the sulfonyl group (Table 1; C11–H11···O2^i^), and the second one between a cyclohexyl H atom and the O atom of the sulfonyl group (Table 1; C12–H12B···O3^ii^). The crystal packing (Fig. 2) is further stabilized by intermolecular C–H···π interactions between a methyl H atom and the benzene ring (Table 1; C10–H10C···Cg^iii^, Cg is the centroid of the C2-C7 benzene ring).

### Experimental

77% 3-chloroperoxybenzoic acid (515 mg, 2.3 mmol) was added in small portions to a stirred solution of 3-cyclohexylsulfanyl-2,5-dimethyl-1-benzofuran (286 mg, 1.1 mmol) in dichloromethane (40 mL) at 273 K. After being stirred at room temperature for 6h, the mixture was washed with saturated sodium bicarbonate solution and the organic layer was separated, dried over magnesium sulfate, filtered and concentrated at reduced pressure. The residue was purified by column chromatography (hexane-ethyl acetate, 4:1 v/v) to afford the title compound as a colorless solid [yield 72%, m.p. 417-418 K; *R*_f_ = 0.66 (hexane-ethyl acetate, 4:1 v/v)]. Single crystals suitable for X-ray diffraction were prepared by slow evaporation of a solution of the title compound in ethyl acetate at room temperature.

### Refinement

All H atoms were positioned geometrically and refined using a riding model, with C–H = 0.95 Å  for aryl, 1.00 Å for methine, 0.99 Å for methylene and 0.98 Å for methyl H atoms, respectively. *U*_iso_(H) = 1.2*U*_eq_(C) for aryl, methine and methylene, and 1.5*U*_eq_(C) for methyl H atoms.

### Crystal data

**Table:**

C_16_H_20_O_3_S	*F*(000) = 624
*M**_r_* = 292.38	*D*_x_ = 1.315 Mg m^−^^3^
Monoclinic, *P*2_1_/*c*	Mo *K*α radiation, λ = 0.71073 Å
Hall symbol: -P 2ybc	Cell parameters from 4533 reflections
*a* = 5.6854 (3) Å	θ = 2.5–27.0°
*b* = 21.2391 (13) Å	µ = 0.22 mm^−^^1^
*c* = 12.3944 (7) Å	*T* = 173 K
β = 99.295 (3)°	Block, colourless
*V* = 1477.01 (15) Å^3^	0.30 × 0.22 × 0.20 mm
*Z* = 4	

### Data collection

**Table:**

Bruker SMART APEXII CCD diffractometer	3228 independent reflections
Radiation source: rotating anode	2477 reflections with *I* > 2σ(*I*)
graphite multilayer	*R*_int_ = 0.035
Detector resolution: 10.0 pixels mm^-1^	θ_max_ = 27.0°, θ_min_ = 1.9°
φ and ω scans	*h* = −6→7
Absorption correction: multi-scan (*SADABS*; Bruker, 2009)	*k* = −21→27
*T*_min_ = 0.643, *T*_max_ = 0.746	*l* = −15→15
13128 measured reflections	

### Refinement

**Table:**

Refinement on *F*^2^	Primary atom site location: structure-invariant direct methods
Least-squares matrix: full	Secondary atom site location: difference Fourier map
*R*[*F*^2^ > 2σ(*F*^2^)] = 0.040	Hydrogen site location: difference Fourier map
*wR*(*F*^2^) = 0.106	H-atom parameters constrained
*S* = 1.05	*w* = 1/[σ^2^(*F*_o_^2^) + (0.0473*P*)^2^ + 0.5178*P*] where *P* = (*F*_o_^2^ + 2*F*_c_^2^)/3
3228 reflections	(Δ/σ)_max_ < 0.001
183 parameters	Δρ_max_ = 0.32 e Å^−^^3^
0 restraints	Δρ_min_ = −0.34 e Å^−^^3^

### Special details

**Table:**

Geometry. All esds (except the esd in the dihedral angle between two l.s. planes) are estimated using the full covariance matrix. The cell esds are taken into account individually in the estimation of esds in distances, angles and torsion angles; correlations between esds in cell parameters are only used when they are defined by crystal symmetry. An approximate (isotropic) treatment of cell esds is used for estimating esds involving l.s. planes.
Refinement. Refinement of F^2^ against ALL reflections. The weighted R-factor wR and goodness of fit S are based on F^2^, conventional R-factors R are based on F, with F set to zero for negative F^2^. The threshold expression of F^2^ > 2sigma(F^2^) is used only for calculating R-factors(gt) etc. and is not relevant to the choice of reflections for refinement. R-factors based on F^2^ are statistically about twice as large as those based on F, and R- factors based on ALL data will be even larger.

### Fractional atomic coordinates and isotropic or equivalent isotropic displacement parameters (Å^2^)

**Table:**

	*x*	*y*	*z*	*U*_iso_*/*U*_eq_	
S1	0.53749 (8)	0.23293 (2)	0.54070 (4)	0.02970 (14)	
O1	0.5649 (2)	0.36998 (6)	0.75483 (10)	0.0354 (3)	
O2	0.7773 (2)	0.21003 (7)	0.56894 (12)	0.0420 (4)	
O3	0.4559 (2)	0.25126 (6)	0.42912 (10)	0.0388 (3)	
C1	0.5009 (3)	0.29734 (8)	0.62295 (14)	0.0296 (4)	
C2	0.3075 (3)	0.34199 (8)	0.60232 (14)	0.0284 (4)	
C3	0.1036 (3)	0.34955 (8)	0.52479 (14)	0.0309 (4)	
H3	0.0678	0.3204	0.4663	0.037*	
C4	−0.0470 (3)	0.39994 (9)	0.53367 (15)	0.0335 (4)	
C5	0.0095 (4)	0.44219 (9)	0.62117 (16)	0.0388 (5)	
H5	−0.0956	0.4763	0.6272	0.047*	
C6	0.2113 (4)	0.43607 (9)	0.69861 (16)	0.0392 (5)	
H6	0.2484	0.4652	0.7571	0.047*	
C7	0.3561 (3)	0.38557 (8)	0.68658 (14)	0.0315 (4)	
C8	0.6487 (3)	0.31577 (9)	0.71474 (14)	0.0332 (4)	
C9	−0.2687 (4)	0.40917 (10)	0.45077 (17)	0.0420 (5)	
H9A	−0.2263	0.4284	0.3846	0.063*	
H9B	−0.3793	0.4368	0.4813	0.063*	
H9C	−0.3449	0.3683	0.4324	0.063*	
C10	0.8718 (3)	0.29097 (11)	0.77813 (16)	0.0409 (5)	
H10A	0.9014	0.2485	0.7525	0.061*	
H10B	0.8573	0.2894	0.8558	0.061*	
H10C	1.0048	0.3186	0.7682	0.061*	
C11	0.3393 (3)	0.17417 (8)	0.57532 (14)	0.0282 (4)	
H11	0.1729	0.1907	0.5573	0.034*	
C12	0.3871 (4)	0.15762 (10)	0.69641 (15)	0.0384 (5)	
H12A	0.5537	0.1430	0.7169	0.046*	
H12B	0.3656	0.1955	0.7404	0.046*	
C13	0.2166 (4)	0.10610 (10)	0.72029 (17)	0.0467 (5)	
H13A	0.2528	0.0944	0.7985	0.056*	
H13B	0.0510	0.1222	0.7054	0.056*	
C14	0.2374 (5)	0.04836 (11)	0.65068 (19)	0.0546 (6)	
H14A	0.1200	0.0164	0.6655	0.066*	
H14B	0.3986	0.0300	0.6704	0.066*	
C15	0.1933 (5)	0.06485 (11)	0.52957 (18)	0.0535 (6)	
H15A	0.0263	0.0789	0.5082	0.064*	
H15B	0.2170	0.0269	0.4863	0.064*	
C16	0.3609 (4)	0.11667 (9)	0.50391 (16)	0.0404 (5)	
H16A	0.3208	0.1286	0.4259	0.049*	
H16B	0.5271	0.1011	0.5172	0.049*	

### Atomic displacement parameters (Å^2^)

**Table:**

	*U*^11^	*U*^22^	*U*^33^	*U*^12^	*U*^13^	*U*^23^
S1	0.0286 (2)	0.0324 (3)	0.0304 (2)	−0.00048 (19)	0.01150 (18)	0.00121 (18)
O1	0.0377 (7)	0.0379 (8)	0.0304 (7)	−0.0085 (6)	0.0050 (6)	−0.0033 (5)
O2	0.0263 (7)	0.0467 (8)	0.0558 (9)	0.0009 (6)	0.0144 (6)	−0.0012 (7)
O3	0.0541 (9)	0.0373 (8)	0.0276 (7)	0.0000 (6)	0.0145 (6)	0.0025 (5)
C1	0.0302 (10)	0.0312 (10)	0.0289 (9)	−0.0056 (8)	0.0097 (7)	0.0009 (7)
C2	0.0311 (9)	0.0276 (9)	0.0286 (9)	−0.0069 (7)	0.0109 (7)	0.0012 (7)
C3	0.0369 (10)	0.0282 (10)	0.0287 (9)	−0.0060 (8)	0.0085 (8)	−0.0001 (7)
C4	0.0364 (10)	0.0280 (10)	0.0370 (10)	−0.0040 (8)	0.0085 (8)	0.0052 (8)
C5	0.0471 (12)	0.0272 (10)	0.0444 (11)	0.0009 (9)	0.0143 (9)	0.0018 (8)
C6	0.0519 (13)	0.0297 (11)	0.0374 (10)	−0.0061 (9)	0.0110 (9)	−0.0067 (8)
C7	0.0349 (10)	0.0320 (10)	0.0284 (9)	−0.0089 (8)	0.0075 (8)	−0.0001 (7)
C8	0.0332 (10)	0.0365 (11)	0.0319 (9)	−0.0077 (8)	0.0116 (8)	0.0033 (8)
C9	0.0413 (12)	0.0382 (12)	0.0458 (12)	0.0000 (9)	0.0047 (9)	0.0088 (9)
C10	0.0300 (10)	0.0582 (13)	0.0337 (10)	−0.0063 (9)	0.0025 (8)	0.0045 (9)
C11	0.0238 (9)	0.0324 (10)	0.0288 (9)	−0.0015 (7)	0.0052 (7)	0.0025 (7)
C12	0.0437 (11)	0.0430 (12)	0.0286 (9)	−0.0096 (9)	0.0064 (8)	0.0049 (8)
C13	0.0515 (13)	0.0547 (14)	0.0339 (10)	−0.0153 (11)	0.0072 (9)	0.0109 (9)
C14	0.0652 (15)	0.0415 (13)	0.0568 (14)	−0.0163 (11)	0.0088 (12)	0.0121 (10)
C15	0.0713 (16)	0.0432 (13)	0.0471 (12)	−0.0193 (11)	0.0131 (11)	−0.0048 (10)
C16	0.0516 (13)	0.0355 (11)	0.0362 (10)	−0.0057 (9)	0.0133 (9)	−0.0034 (8)

### Geometric parameters (Å, °)

**Table:**

S1—O2	1.4368 (13)	C9—H9C	0.9800
S1—O3	1.4395 (13)	C10—H10A	0.9800
S1—C1	1.7385 (18)	C10—H10B	0.9800
S1—C11	1.7797 (17)	C10—H10C	0.9800
O1—C8	1.369 (2)	C11—C12	1.523 (2)
O1—C7	1.382 (2)	C11—C16	1.525 (3)
C1—C8	1.358 (3)	C11—H11	1.0000
C1—C2	1.443 (3)	C12—C13	1.522 (3)
C2—C7	1.390 (2)	C12—H12A	0.9900
C2—C3	1.391 (3)	C12—H12B	0.9900
C3—C4	1.386 (3)	C13—C14	1.515 (3)
C3—H3	0.9500	C13—H13A	0.9900
C4—C5	1.404 (3)	C13—H13B	0.9900
C4—C9	1.505 (3)	C14—C15	1.522 (3)
C5—C6	1.378 (3)	C14—H14A	0.9900
C5—H5	0.9500	C14—H14B	0.9900
C6—C7	1.375 (3)	C15—C16	1.522 (3)
C6—H6	0.9500	C15—H15A	0.9900
C8—C10	1.477 (3)	C15—H15B	0.9900
C9—H9A	0.9800	C16—H16A	0.9900
C9—H9B	0.9800	C16—H16B	0.9900
			
O2—S1—O3	118.14 (8)	C8—C10—H10C	109.5
O2—S1—C1	108.80 (9)	H10A—C10—H10C	109.5
O3—S1—C1	107.46 (8)	H10B—C10—H10C	109.5
O2—S1—C11	108.47 (8)	C12—C11—C16	111.65 (16)
O3—S1—C11	107.62 (8)	C12—C11—S1	112.35 (12)
C1—S1—C11	105.67 (8)	C16—C11—S1	107.96 (12)
C8—O1—C7	107.10 (14)	C12—C11—H11	108.2
C8—C1—C2	108.03 (16)	C16—C11—H11	108.2
C8—C1—S1	126.98 (15)	S1—C11—H11	108.2
C2—C1—S1	124.96 (13)	C13—C12—C11	109.85 (16)
C7—C2—C3	118.99 (17)	C13—C12—H12A	109.7
C7—C2—C1	104.56 (16)	C11—C12—H12A	109.7
C3—C2—C1	136.45 (16)	C13—C12—H12B	109.7
C4—C3—C2	119.42 (17)	C11—C12—H12B	109.7
C4—C3—H3	120.3	H12A—C12—H12B	108.2
C2—C3—H3	120.3	C14—C13—C12	111.12 (17)
C3—C4—C5	119.18 (18)	C14—C13—H13A	109.4
C3—C4—C9	120.43 (17)	C12—C13—H13A	109.4
C5—C4—C9	120.39 (18)	C14—C13—H13B	109.4
C6—C5—C4	122.59 (18)	C12—C13—H13B	109.4
C6—C5—H5	118.7	H13A—C13—H13B	108.0
C4—C5—H5	118.7	C13—C14—C15	111.09 (18)
C7—C6—C5	116.34 (18)	C13—C14—H14A	109.4
C7—C6—H6	121.8	C15—C14—H14A	109.4
C5—C6—H6	121.8	C13—C14—H14B	109.4
C6—C7—O1	126.21 (16)	C15—C14—H14B	109.4
C6—C7—C2	123.48 (18)	H14A—C14—H14B	108.0
O1—C7—C2	110.31 (16)	C14—C15—C16	111.32 (18)
C1—C8—O1	110.00 (16)	C14—C15—H15A	109.4
C1—C8—C10	134.80 (19)	C16—C15—H15A	109.4
O1—C8—C10	115.20 (16)	C14—C15—H15B	109.4
C4—C9—H9A	109.5	C16—C15—H15B	109.4
C4—C9—H9B	109.5	H15A—C15—H15B	108.0
H9A—C9—H9B	109.5	C15—C16—C11	110.25 (16)
C4—C9—H9C	109.5	C15—C16—H16A	109.6
H9A—C9—H9C	109.5	C11—C16—H16A	109.6
H9B—C9—H9C	109.5	C15—C16—H16B	109.6
C8—C10—H10A	109.5	C11—C16—H16B	109.6
C8—C10—H10B	109.5	H16A—C16—H16B	108.1
H10A—C10—H10B	109.5		
			
O2—S1—C1—C8	−13.71 (19)	C3—C2—C7—O1	179.92 (14)
O3—S1—C1—C8	−142.70 (16)	C1—C2—C7—O1	0.39 (18)
C11—S1—C1—C8	102.59 (17)	C2—C1—C8—O1	−0.52 (19)
O2—S1—C1—C2	164.31 (14)	S1—C1—C8—O1	177.76 (12)
O3—S1—C1—C2	35.31 (17)	C2—C1—C8—C10	179.59 (19)
C11—S1—C1—C2	−79.39 (16)	S1—C1—C8—C10	−2.1 (3)
C8—C1—C2—C7	0.08 (18)	C7—O1—C8—C1	0.76 (19)
S1—C1—C2—C7	−178.25 (13)	C7—O1—C8—C10	−179.33 (15)
C8—C1—C2—C3	−179.33 (19)	O2—S1—C11—C12	59.13 (15)
S1—C1—C2—C3	2.3 (3)	O3—S1—C11—C12	−171.99 (13)
C7—C2—C3—C4	−0.5 (2)	C1—S1—C11—C12	−57.40 (15)
C1—C2—C3—C4	178.88 (18)	O2—S1—C11—C16	−64.43 (15)
C2—C3—C4—C5	−0.2 (3)	O3—S1—C11—C16	64.46 (14)
C2—C3—C4—C9	179.96 (16)	C1—S1—C11—C16	179.05 (13)
C3—C4—C5—C6	0.7 (3)	C16—C11—C12—C13	−57.1 (2)
C9—C4—C5—C6	−179.43 (18)	S1—C11—C12—C13	−178.58 (14)
C4—C5—C6—C7	−0.6 (3)	C11—C12—C13—C14	56.9 (2)
C5—C6—C7—O1	−179.30 (16)	C12—C13—C14—C15	−56.7 (2)
C5—C6—C7—C2	−0.2 (3)	C13—C14—C15—C16	55.8 (3)
C8—O1—C7—C6	178.52 (17)	C14—C15—C16—C11	−55.3 (3)
C8—O1—C7—C2	−0.71 (18)	C12—C11—C16—C15	56.4 (2)
C3—C2—C7—C6	0.7 (3)	S1—C11—C16—C15	−179.62 (15)
C1—C2—C7—C6	−178.87 (17)		

### Hydrogen-bond geometry (Å, °)

**Table:**

Cg is the centroid of the C2–C7 benzene ring

**Table:**

*D*—H···*A*	*D*—H	H···*A*	*D*···*A*	*D*—H···*A*
C11—H11···O2^i^	1.00	2.31	3.273 (2)	161
C12—H12B···O3^ii^	0.99	2.57	3.443 (2)	146
C10—H10C···Cg^iii^	0.99	2.75	3.556 (2)	140

Symmetry codes: (i) *x*−1, *y*, *z*; (ii) *x*, −*y*+1/2, *z*+1/2; (iii) *x*+1, *y*, *z*.
